# A Look at Collaborative Service Provision: Case for Cosmetic Surgery Medical Tourism at Korea for Chinese Patients

**DOI:** 10.3390/ijerph182413329

**Published:** 2021-12-17

**Authors:** Jungkun Park, Hoang Tran Phuoc Mai Le, Eklou R. Amendah, Dongyoup Kim

**Affiliations:** 1 School of Business, Hanyang University, 222 Wangshimni-ro, Seongdong-gu, Seoul 04763, Korea; viroid2007@gmail.com (J.P.); letranphuocmaihoang@gmail.com (H.T.P.M.L.); 2 School of Business, University of Southern Maine, Portland, ME 04104, USA; eklou.amendah@maine.edu; 3 School of Business, Gachon University, 1342 Seongnam-daero, Sujeong-gu, Seongnam 13120, Korea

**Keywords:** Chinese patient, medical facilitators, Korean hospitals, cosmetic surgery, medical tourism

## Abstract

Consumers admiring the beauty standards of other countries are approaching cosmetic surgery medical tourism. This study examines the roles of hospitals and facilitating agents as the main entities of cosmetic surgery medical tourism. 334 Chinese patients who underwent cosmetic surgery in Korea were collected and structural equation modeling is used to analyze the data. The results show that a hospital’s service quality in terms of tangibles, assurance, and empathy affect customers’ attitudes toward medical tourism for cosmetic surgery, which in turn, influences satisfaction with medical tourism. More importantly, facilitating agents’ service quality moderates the effects of hospitals’ service quality dimensions on service satisfaction. Findings extend the existing literature on medical tourism by identifying the roles of hospitals and facilitating agents to enhance customers’ attitudes and satisfaction with respect to collaborative service provision. Moreover, this research provides the first empirical evidence for the facilitating agents’ role in determining satisfaction with medical tourism.

## 1. Introduction

People undergo physical modifications and cosmetic surgeries in pursuit of beauty. This induces a transformation of the tourism industries according to the desirability of physical appearances within a particular society. With the global development of the information technology system, consumer preferences have been focused on global beauty standards rather than domestic ones [1]. As the ripple effect of cultural contents increases, the desirable social appearance standard becomes easy to change; this has rapidly expanded the business of medical tourism for cosmetic surgery [2]. Besides providing therapeutic services, medical tourism for cosmetic surgery has been positioned as a new investment field and delivering high added value to various countries [3]. Further, a collaboration between various business entities is encouraged for the effective provision of services in the structure of the service attributes [4,5].

It is worth noting that cosmetic surgery medical tourism is considered to be popular among consumers seeking overseas treatment [6]. According to the International Society of Aesthetic Plastic Surgery, breast augmentation using silicone implants has emerged as the most famous treatment worldwide, followed by liposuction, abdominoplasty, and rhinoplasty that have been adopted by 1.8 million (15.8%), 1.7 million (15.0%), 0.9 million (8.1%), and 0.8 million (7.2%) individuals, respectively [7]. This trend in the popularity of cosmetic surgery is reflected among Chinese patients as well. It is estimated that out of approximately 3 million Chinese consumers who visited South Korea in 2014, approximately one million were expected to be engaged in medical tourism for cosmetic surgery [4]. Considering the great success of Korean cultural content in China, many Chinese consumers are likely to spend a significant amount of money on cosmetic surgery to acquire a South Korean appearance [4,8,9]. Korean cosmetic surgery, called “Korean Look”, includes a set of facial surgery performed on the eyes, cheekbones, jawbones, and nose tip [4]. In contrast to simple “westernization”, it was characterized by leveraging Korea’s cultural force. In addition, the technological skills of surgeons in a competitive environment from medical schools highlighted the cosmetic surgery industry, and support from the health insurance aspect also acted as an external environmental factor.

Existing literature has conceptualized the service provision of medical tourism as follows. According to a conceptual model proposed from the consumer’s perspective, service delivery in medical tourism may be expressed in two sequential stages [10]. In the first stage of decision-making regarding medical tourism, consumers decide a destination, and, in the second stage, they choose a healthcare facility within the destination, such that they can select and evaluate the service attributes that are desirable to them [11]. In addition, the push and pull dichotomization of factors influencing consumer motivation in medical tourism has contributed toward distinguishing the core and peripheral service attributes for incorporated services [12,13]. Following the dichotomization, the consumers of medical tourism usually regard service attributes that are provided by hospitals as core services, and relatively perceived the factors related to “tourism” in a peripheral manner. The quality assessment for each service attribute was found to have a positive effect on service satisfaction; moreover, since the services are provided in independent stages, their positive effect was expressed through a parallel path [14].

Unlike the previous conceptualization of medical tourism service providers, the service areas that were treated as peripheral are now being provided by independent facilitating agents; their role has become as important as that of hospitals (Park et al., 2020). For this reason, hospitals and facilitating agents no longer provide discrete services and have formed a mutual collaboration or nested structure. According to the conceptual model proposed by Karadayi-Usta and Serdarasan (2020), which describes the supply side of medical tourism, the medical tourism service provision structure can be explained in various ways depending on the role of the facilitating agent, and the role of the facilitator is interrelated [3]. Due to this change, consumers should consider the interaction between the two entities while assessing overall service satisfaction with medical tourism. In this regard, this study aims to provide empirical evidence to show the interactive manner of service provision by hospitals and facilitating agents in cosmetic surgery medical tourism. Specifically, the research questions that seek answers in this study are as follows: (1) which service quality dimensions of hospitals mainly affect patients’ attitudes and satisfaction with cosmetic surgery medical tourism? (2) Does facilitating agents’ service quality have a moderating effect on the relationship between hospital service quality on patients’ attitude and satisfaction with cosmetic surgery medical tourism?

This study determines the touchpoints with patients and the quality of services provided at each point by the facilitating agents and healthcare institutions. In the field of service marketing, the control of touchpoint experiences by service providers has the potential to contribute toward an effective overall experience for the customer; this is because they expect seamless experiences when healthcare services are delivered [15]. It is important for the patients who travel to a foreign country seeking cosmetic surgery to have a positive encounter with the direct and indirect interactions between the service providers involved in the incorporated service. Therefore, this study shows how the quality of service provided by core and peripheral service providers interactively builds up customer service satisfaction in the context of medical tourism. This analysis on the impact of the collaboration between the two entities on the medical tourism for cosmetic surgery contributes to the existing literature by empirically examining the service provision of the supply side and how the detailed service dimension should be planned, in addition to focusing on the influence of service quality that has been observed in prior studies.

A conceptual model is proposed based on the literature review to investigate the relationships among the constructs related to the research questions. The dataset was collected from patients who use facilitating agents to implement medical tourism for cosmetic surgery in Korean hospitals. In the following section, the measurement test and the hypothesis testing by structural equation modeling are presented. Finally, we conclude the study by providing theoretical and practical implications, limitations, and future research directions.

## 2. Review of Literature and Hypotheses Development

### 2.1. Provision of Medical Tourism Services

The international healthcare system has recently registered an increase in the medical tourism service worldwide [4,16]. Related studies in this field have attempted to understand this phenomenon and investigate individuals’ motivations behind seeking such services overseas [4,17,18]. Consumer motivation has been broadly investigated through the pull and push factor dichotomization of medical tourism. For instance, Cormany (2013) has explained the reasons behind the rise in medical tourism in terms of high healthcare expenses, low health insurance coverage, improvement of healthcare technology, and a systematic decrease of the healthcare workforce in countries that see a significant exodus of their patients toward other countries [19].

This investigation of pull and push factors, combined with a two-stage model [10,11]. describing the service provision structure of medical tourism has helped distinguish the core and peripheral service attributes within medical tourism. It has divided consumers’ decision-making regarding medical tourism into two sequential stages and identified the primary factors observed in each stage. The first stage considers the choice of country for treatment, while the second stage is dedicated toward the decision-making for an appropriate selection of treatment institutions. Consumers put more effort into selecting the service attributes delivered by healthcare institutions in the sequential service provision separated by the two-stage model; they have regarded it as a more critical aspect while evaluating the service satisfaction for medical tourism [13]. From this point of view, the framework for core and peripheral service attributes has indicated the first stage as a relatively peripheral service [20,21].

However, the current medical tourism service is changing from the existing discrete two-stage model to a collaborative and nested structure. In fact, the services provided by healthcare institutions and facilitating agents have combined to create a complex web of services. Since the area in charge of “tourism” in the first stage, which was mainly recognized as the role of assisting core service attributes in charge of medical facilities, has expanded, the supply chain of medical tourism has diversified with the collaboration of the two entities. Accordingly, the newly proposed supply chain of the medical tourism service has emphasized the importance of coordination between the two stages with a focus on the role played by a facilitating agent in collaborative service provision [3].

It is worth noting that there are many actors involved in the delivery of cosmetic surgery services. With a growing demand for medical tourism, the number of facilitating agents for healthcare services is increasing. The agents’ role in managing medical tourism is critical, especially in the case of providing services to foreign patients. In general, agents play four types of differentiated roles: travel planner, medical case manager, destination medical concierge, and marketing and web portal developer [5]. They play an important role in connecting foreign patients with local hospitals and provide direct and indirect services to improve their experience [4,5]. Such direct services are inherent to medical procedures, whereas the indirect services pertain to providing information related to the logistics of transporting the patients to and from the place of care and recovery. Certain examples of direct services include the provision of translating medical procedures to consumers, recovery methods, and selecting medical facilities. Indirect services are related to that of travel visas, transportation, and referral services [22].

### 2.2. Service Quality in Medical Tourism

To measure service performance, Parasuraman et al. (1988) proposed five dimensions: tangibles, reliability, responsiveness, assurance, and empathy [23]. Tangibles pertain to the factors considering the appearance of the facility and the service structure. Empathy refers to the extent to which an individual service provider shows care and individualized attention. Assurance reflects on the providers’ extensive knowledge about the service delivered and the extent to which they can gain the receiver’s trust through the effective and efficient conveyance of service information. Reliability refers to the accuracy of service performance, whereas Responsiveness refers to providers’ willingness to grant such services in accordance with consumers’ expectations [23].

Babakus and Boller (1992) used SERVQUAL to conceptualize the technical and functional aspects of healthcare services [24]. The technical aspect represents the appropriateness of diagnosis, treatments, and procedures, whereas the functional aspect illustrates how the service was being delivered to patients. It must be observed that the functional aspect of healthcare services is the only way of evaluating the overall services, especially for the patients who lack the technical knowledge to assess the accuracy of their diagnosis and treatments; moreover, even healthcare practitioners who have sufficient technical knowledge cannot fully guarantee a positive outcome for certain treatments. Similarly, as cosmetic surgery is not a treatment performed for a cure, patients do not have to assess the services based on technical knowledge. Therefore, the service quality of cosmetic surgery is evaluated by considering the functional aspect of SERVQUAL, which exhibits consumers’ satisfaction with the service delivered.

Facilitating agents form an integral part of medical tourism. Overall, their services range from providing medical referrals and scheduling services to making travel arrangements [22]. In a comprehensive study, Gan and Frederick (2018) identified four types of facilitators in medical tourism [25]. (1) Self-facilitator is defined as an individual who knows about the healthcare services provided in foreign hospitals. This type of facilitator includes local networks such as friends and relatives who stay abroad and can share useful information with respect to treatment decisions [26,27]. (2) Agents in medical tourism play the role of middlemen and help patients to save a considerable amount of time and money, especially in the context of research and travel planning [25]. (3) Health insurers or employers offer incentives to seek effective medical treatments in foreign countries with cheaper prices as compared to the domestic market [25,28]. (4) Domestic doctors transfer certain patients to foreign hospitals for availing better procedures, effective care systems, and sophisticated health equipment [29].

In this context, Dalstrom (2013) has classified the services provided by facilitating agents into three categories: full, referral, and individual services [22]. A full-service medical agent provides comprehensive services that include travel details, local accommodation and transportation, physician referral, translation service, and post-surgery care. The role of the referral service facilitator is to connect the patient to potential medical providers. However, this agent does not provide travel logistics and post-surgery care services [22]. Finally, individual service facilitators mainly advertise the services of the local medical institution to medical tourism consumers and act as translators during the procedures [30].

Consumers assess the service quality in medical tourism through the dimensions of SERVQUAL, which are influenced by the services offered by facilitating agents. Patients receiving medical treatment in foreign countries wish to obtain different types of services compared to domestic treatments. For example, patients expect to receive assurance while waiting for a medical procedure to reduce anxiety, especially when they are uncertain about the outcome. For this reason, facilitators try to provide assurance by presenting their extensive knowledge about logistics. Regarding the reliability dimension, the patients seeking treatment overseas may not have control over the outcome of the procedure; therefore, they expect to receive reliable, up-to-date, prompt, and certain information from the facilitating agents. Finally, since most facilitating agents provide online services [22,31], patients can rely on the user-friendly aspect of their websites and evaluate the services accordingly.

### 2.3. Cosmetic Surgery Medical Tourism by Chinese Patients

Cosmetic surgery is a lucrative and rising enterprise that has been broadly defined as an elective procedure adopted by consumers for changing their physical appearance [32,33]. This has been especially observed in the US, China, and Brazil [34]. Tiggemann (2013) and Fredrickson and Roberts (1997) have observed that the motivation behind seeking cosmetic surgery lies in appearance and self-scrutiny [35,36]. Certain studies have used more complicated concepts to explain the motivation for cosmetic surgery, such as self-objectification and tripartite influence [32,37]. Self-objectification theorists conclude that social pressure compels consumers to idealize socially desirable physical characteristics. The tripartite influence concept posits that unrealistic societal norms conveyed by media, friends, and family members pressure consumers into being dissatisfied with their physical appearance [38,39].

Prior studies have predicted the boom of China’s cosmetic surgery industry based on their notion of youthful beauty or “nennu” [40,41]. Young women in China are responsible for more than 70% of its cosmetic surgeries and they primarily seek to modify facial expressions. The most popular procedures among Chinese consumers include double-eyelid surgery [40], rhinoplasty, and cheekbone and jawbone shaving [9]. It is also observed that such types of procedures are popular among Chinese men as well as women. Since Chinese consumers undergo cosmetic surgery as a response to social norms, social pressure, and culturally prescribed physical characteristics, it is critical for them to obtain good surgical outcomes. This encourages consumers to select their health institution carefully. When consumers decide to receive cosmetic surgeries abroad, their lack of such knowledge compels them to rely on the service provided by facilitating agents.

### 2.4. The Effect of Service Quality Dimensions on Attitude and Satisfaction

Uncertainty about the outcome of medical procedures and lack of knowledge about South Korean surgery practices make it difficult for Chinese consumers to assess the quality of the procedure due to the tangible nature of the service. However, prior studies have observed that this tangible nature acts as a significant factor in the patient’s evaluation of the healthcare service [42,43]. The environment in which the procedure is performed, the size of the caregiving personnel, and the amenities available at the institution providing such care tend to affect the patient’s perception of the service [44,45]. In this context, we suggest the following hypothesis:

**Hypothesis** **1.** *Customers’* *perceived tangibility in service quality is positively related to their attitude toward medical tourism for cosmetic surgery.*

South Korea accounts for more than 6% of the medical tourism market in Asia. The Korean government declared medical tourism as one of the most significant branches of the national economy in 2009 [46,47]. Consequently, the government invested in hospitals, specialty clinics, advanced medical technology, and state-of-the-art healthcare facilities [48,49]. It also started issuing medical tourism visas that made it easier for healthcare institutions to advertise cosmetic surgery [46].

The boom experienced by the South Korean cosmetic industry is partly accountable to the reputation and the quality of services provided by its cosmetic surgeons [4,48]. The government has invested in quality education for its national surgeons to develop and improve their skills [46,50]. Guiry and Vequist (2015) suggested that the competence of Korean surgeons could be utilized as a marketing tool by highlighting their qualifications and educational background [46].

Chinese patients seeking cosmetic surgery visit Korea after recognizing their high reputation in this field. Jin (2015) revealed that 60% of medical tourists in South Korea are from China. Moreover, South Korean surgeons who are invited to perform such surgeries in China place a high premium on their professional services [4,51]. Further, in terms of the reliability of cosmetic surgery, service quality can improve the attitude of consumers. Therefore, we hypothesize as follows:

**Hypothesis** **2.** *Customers’ perceived reliability in service quality is positively related to their attitude toward medical tourism for cosmetic surgery.*

Responsiveness in service quality is exhibited through providers’ willingness to assist customers and in providing prompt and adequate services [23]. Chinese patients tend to be demanding, confused, and unrealistic when they visit Korea in the context of medical tourism [4]. However, Korean cosmetic surgeons respond to their demands by discussing the process, risks, and outcomes that are associated with certain procedures [4]. Therefore, the hospital’s accurate and prompt response to patients’ concerns is important. It must be noted that patients appreciate the promptness of their service in an environment where a successful outcome is not completely guaranteed, such as in the case of cosmetic surgery. Therefore, we hypothesize as follows:

**Hypothesis** **3.** *Customers’ perceived service quality in the context of responsiveness is positively related to their attitude toward medical tourism for cosmetic surgery.*

Parasuraman et al. (1988: p. 23) referred to assurance as the “knowledge and courtesy of employees and the ability to convey trust and confidence” [23]. In cosmetic surgery, where a completely positive outcome is not guaranteed, the question of how hospitals can increase trust and confidence in consumers is of primary concern. It is crucial because consumers tend to rely on assurance to inform their purchase decision [52]. By showing a positive attitude towards customers, that is, assuring customers, paying close attention, and demonstrating knowledge about their services, service providers build consumers’ confidence. Furthermore, research shows that trust plays an important role in consumers’ decisions to establish a distinctive destination personality [53,54]. Therefore, we hypothesize as follows:

**Hypothesis** **4.** *Customers’ perceived service quality in assurance is positively related to their attitude toward medical tourism for cosmetic surgery.*

Empathy is broadly defined as the ability to think in the other person’s shoes or feel pain [55]. In the context of healthcare, empathy is conceptualized as the provider’s ability to comprehend the patient’s situation, relate to the patient’s feelings, and check whether the patient understands the situation accurately to deliver an effective and efficient treatment [56].

Empathy is characterized as caring for and paying individualized attention to customers while delivering service [23]. It has been established as one of the strongest determinants of service quality [57]. Especially in the context of cosmetic services, Chinese patients experience considerable social pressure to change their appearance to conform to social norms [39,58]. Moreover, Holliday et al. (2017) found that many Chinese women undergo cosmetic surgery for economic reasons. Finding a well-paid job now requires “good” or “desirable” looks [4,59]. These social and economic pressures, coupled with the uncertainty about the outcome of the surgery, affect the patient’s emotional and psychological well-being. Therefore, emotions experienced by the patient before, during, and after surgery must be managed well. We, therefore, hypothesized that:

**Hypothesis** **5.** *Customers’ perceived service quality in empathy is positively related to their attitude toward medical tourism* *for cosmetic surgery.*

Self-perception theory indicates that past behaviors directly influence attitudes, with little mediation of cognitive activity [60]. Moreover, the level of satisfaction with the performance of these behaviors can affect the impact of past engagement on attitudes. Hence, higher satisfaction from past engagement will cause favorable attitudes compared to those who experience lower satisfaction [61]. Satisfaction is an intrinsic positive cognition rather than an extrinsic one and follows a behavior that fulfills individual expectations [62]. The expectancy disconfirmation model proposed by Oliver (1980) asserts that when behavior conforms to expectations, expectancy confirmation is observed and consequently, it positively affects satisfaction [63,64].

**Hypothesis** **6.** *Customers’ attitude toward the cosmetic surgery medical tourism service is positively related to their satisfaction with the service.*

### 2.5. Moderating Effect of Facilitating Agents’ Service Quality

Reasons for using agents are unfamiliarity with the details of medical care at a specific medical facility and the convenience of avoiding the logistics involved in complicated medical procedures [65]. Medical facilitating agents and hospitals know that the services they provide to foreign patients must meet certain quality standards. The services offered by the hospitals and facilitating agents are quite different and unrelated to each other. A patient’s assessment of service quality sometimes considers the services provided by both the hospitals and facilitating agents. In fact, Guiry and Vequist (2015) considered medical tourism as a destination brand personality, which refers to “the set of human characteristics associated with a destination” [46,53] (p. 128). This concept elucidates the tourists’ understanding of places and the identity attached to them [53]. In particular, the study emphasized that medical tourists perceive Korea as a major cosmetic surgery destination due to its distinctive services, regardless of the specific institutions. Chinese consumers evaluate alternative medical tourism destinations by combining the services offered by both the hospitals and facilitator agents. Therefore, discrepancies in service quality between hospitals and facilitating agents may affect the overall perception of service quality.

Facilitating agents also influence hospital responsiveness on consumer attitudes. Altin et al. (2013) asserted that travel intermediaries or third-party entities who plan and execute the entire journey motivate medical travel [66]. Accordingly, Mohamad et al. (2012) described medical travel facilitators as the most essential information gatekeepers in the travel purchase decision-making process [67]. Mohamad et al. (2012) found that facilitators encourage both patients and healthcare service providers to engage in medical tourism and showed that 61% of respondents agreed on the fact that facilitators contribute to their decision on pursuing treatment in another country [67]. Facilitators further shape the patient’s impression of the country’s healthcare systems and their intention of revisiting it. Although facilitators do not provide direct medical care, they interact with the patient at all levels, which requires empathy. Therefore, we hypothesize the effect of facilitating agents’ service quality as follows:

**Hypothesis** **7.** *Customers’ perceived service quality of facilitating agents moderates the effect of hospitals’ service quality on their attitude toward medical tourism for cosmetic surgery.*

**Figure 1:**
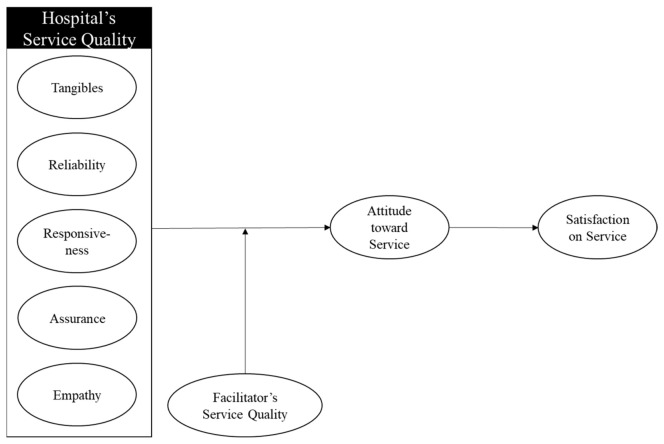
Conceptual framework.

Figure 1 depicts how hospital service quality including tangibles, reliability, responsiveness, assurance, and empathy affect attitude toward service and satisfaction with the service, as a structural equations model. The facilitator’s service quality was described as a moderator of the relationship between hospital service quality and customer’s attitude.

## 3. Methodology

### 3.1. Data Collection

Data were collected from the inbound patients who decided to obtain medical tourism services for cosmetic surgery in Korea in the following way. First, with the help of the Korea Tourism Organization, we accessed five cosmetic surgery hospitals that performed surgeries on the largest number of foreigners as of 2019. The five hospitals selected for data collection were mainly distributed in Seoul, Korea, especially in the Gangnam area. With the hospitals’ cooperation, consumers of Chinese medical tourism were asked to respond to a paper-and-pencil questionnaire in English. To make sure the respondents’ understanding of the questionnaire content, an interpreter who is fluent in both Chinese and English was recruited to support respondents during the time when they conduct the survey.

Accordingly, 334 Chinese consumers’ responses were collected with the purpose of investigating the perception of their overall medical travel experience. We collected only the records of Chinese patients who voluntarily expressed their intention to participate, and incomplete responses were excluded. The questionnaire was structured in the following order. First, the respondents commented on the quality of service provided by the two entities, that is, the hospitals and tourism facilitators, respectively. Second, they evaluated their attitude and satisfaction after obtaining medical tourism service sequentially. Finally, the participants recorded their demographic information and were asked to answer questions regarding the availability of health insurance and prior experience of medical tourism.

A total of 20 and 18 items were used for measuring the quality of service provided by hospitals and facilitating agents, respectively. The measurement items of the five SERVQUAL dimensions (tangibles, assurance, empathy, reliability, and responsiveness) adopted by Parasuraman et al. (1988) have been modified and used to analyze the services provided by the hospital and facilitator [23]. The SERVQUAL developed by Parasuraman et al. (1988) consists of 22 measurement items, but that applied to hospitals in medical tourism services has been mainly measured with 15 items [23,68,69]. SERVQUAL of outpatient department service in medical tourism was formed by adding five and three more items for hospital and facilitator, respectively [70]. Detailed information about the measurement items has been presented in Appendix A.

This study was drafted according to the STROBE statement [71].

### 3.2. Sample Description

Female participants accounted for 73.4% of the respondents. Since 35.6% of the participants were aged between 30 and 39, they represented the largest age group, followed by those aged between 18 and 29 (30.8%), and those aged between 40 and 49 (21.3%). Consequently, the sample comprised participants who were young and of working age. Most participants received an annual income ranging between $25,000 to $39,999 (30.2%) and $40,000 to $54,999 (29%). Moreover, 47.6% of the participants were married, unlike 33.8% of them. While 53.9% of the participants had health insurance, 59.3% of the participants confided that they had never traveled abroad for medical services. Further, 37.7% of participants answered that it was their first experience in medical tourism. The socio-demographic characteristics of the participants are represented in Table 1.

### 3.3. Data Analysis

We test the perceived dimensionality of hospitals’ and facilitator’s service quality prior to investigating their effects on attitude and satisfaction in the context of medical tourism for cosmetic surgery. Accordingly, we identify whether there exists a significant difference in consumers’ perception about the dimensionality of service quality for hospitals and facilitators compared with the existing SERVQUAL. In this study, an exploratory factor analysis was adopted to test the dimensionality of service quality for service collaborative partners. The resultant factors were extracted by applying varimax rotation.

We conducted CFA on the constructs suggested in the research model. By using AMOS 21, the standardized loadings of the observed variables of each construct were estimated. The AVE, MSV, and CR of each latent variable were calculated accordingly, which were used to test convergent validity and reliability. In addition, the correlation between the latent variables and the square root of AVE were compared to test discriminant validity.

Subsequently, a structural equation model was constructed to test the hypotheses proposed in this study. First, we tested the primary effect of a hospital’s service quality on consumers’ attitude and service satisfaction toward cosmetic surgery in the context of medical tourism to verify the influence of the core service providers. Second, with a multi-group analysis, we tested the role of the facilitator’s service quality in the relationship between the hospital’s service quality, customer’s attitude, and service satisfaction. By dividing participants into two groups according to the perceived service quality of the facilitator, we investigate whether there exists a difference in the SERVQUAL dimensions that significantly affect service evaluation.

## 4. Results

### 4.1. EFA on the Service Quality of Hospitals and Facilitators

The results of the EFA on the hospital’s service quality indicated that 20 items were categorized into five factors, which accounted for 71.28% of the total variance. Both the KMO index and Barlett’s test were significant (KMO measure = 0.962; Bartlett’s test: χ^2^_190_ = 4412.821, *p* < 0.001). In the case of the rotated factor solution, only the loadings that were higher than 0.40 are considered, as shown in Table 2. The five observed factors are tangibles, reliability, responsiveness, assurance, and empathy, which are equivalent to the existing SERVQUAL dimensionality. Cronbach’s alpha for each dimension also exceeded 0.7, indicating acceptable reliability for all the factors.

Different from the hospital’s dimensionality, the results of EFA on the facilitator showed that 18 items of SERVQUAL were categorized into a single factor that accounted for 58.33% of the total variance. For the rotated solution, both the KMO index and Barlett’s test were significant (KMO measure = 0.960; Bartlett’s test: χ^2^_153_ = 4349.815, *p* < 0.001). The EFA on facilitator’s SERVQUAL indicates that Chinese consumers visiting Korea for cosmetic surgery in the context of medical tourism holistically assess the service quality of the facilitator. Detailed information about the abbreviation has been presented in Abbreviations part.

### 4.2. CFA on the Focal Constructs

The results of CFA performed before testing the relationship between the focal constructs presented in the research model are as follows. The CFA model for the seven constructs, including attitude and satisfaction for cosmetic surgery tourism service, showed a satisfactory goodness-of-fit (χ^2^_277_ = 400.853, *p* < 0.001; GFI = 0.92; NFI = 0.94; CFI = 0.98; RFI = 0.92; IFI = 0.98; TLI = 0.98; RMSEA = 0.037). According to the results, the AVE values of service quality dimensions for the five hospitals, attitude, and satisfaction exceeded 0.5, which is an acceptable level of convergent validity. In addition, the composite reliability of each construct ranged from 0.82 to 0.89, ensuring the reliability of measurement items of each construct.

To test the discriminant validity of the focal constructs, MSV, square root of AVE, and correlations were calculated with different constructs. All the correlation values were accepted as they are smaller than 0.8. In addition, all MSV values were smaller than AVE and the square roots of AVE were greater than the correlation between every pair of latent variables, which ensures discriminant validity [72]. Table 3 shows the results of CFA for focal constructs.

### 4.3. Structural Equation Model

For the structural equation model, the goodness-of-fit indices showed a satisfactory level to test the hypothesized paths. The Chi-square value was acceptable (χ^2^_282_ = 429.774, *p* < 0.001), while the GFI, NFI, CFI, RFI, IFI, and TLI values were greater than 0.9. However, RMSEA was less than 0.05 (GFI = 0.91; NFI = 0.93; CFI = 0.98; RFI = 0.92; IFI = 0.98; TLI = 0.97; RMSEA = 0.040).

The result of the structural equation model shows that four paths (H1, H4, H5, and H6) were supported. As shown in Table 4, hospital tangibles, assurance, and empathy significantly and positively affected customers’ attitudes toward cosmetic surgery tourism service, which in turn affect customers’ service satisfaction. Particularly, assurance had the most significant effect as compared to tangibles and empathy of SERVQUAL (β = 0.415). Different from the three SERVQUAL dimensions, hospitals’ reliability and responsiveness did not show a significant effect on attitudes toward the tourism service and did not support H2 and H3. The results also showed a strong positive impact of customers’ attitudes toward cosmetic surgery tourism service on service satisfaction (β = 0.808).

### 4.4. Moderating Effect of Facilitators’ Service Quality

**Figure 2:**
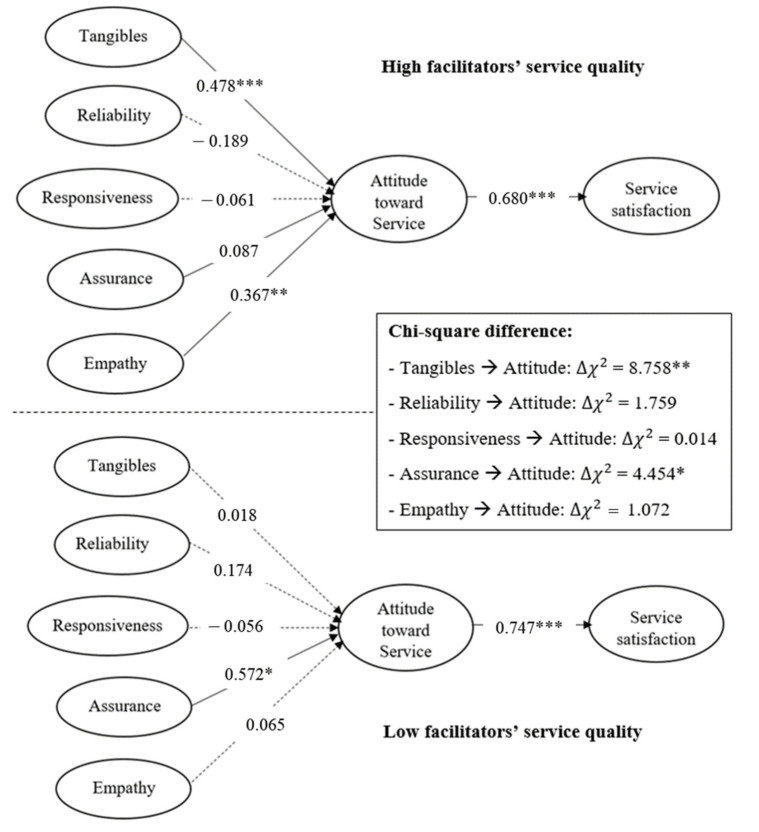
Results of SEM for high and low facilitators’ service quality (*n* = 162 and *n* = 172) respectively). Note: *p* * < 0.05, *p* ** < 0.01, *p* *** < 0.001.

To test the moderating effect of the level of facilitators’ service quality, the participants were divided into two groups according to their assessment of the facilitators’ service quality: high (*n* = 162) and low (*n* = 172). Before performing the multi-group analysis, we confirmed is the presence of a model-level difference between the two groups. The chi-square test for the invariance test indicated that there exists a significant difference between the unconstrained and constrained models (∆χ^2^ = 11.22, ∆df = 5; *p* = 0.047). Therefore, a multi-group analysis was further performed to investigate which hypothesized paths were moderated by the level of facilitators’ service quality and showed a satisfactory goodness-of-fit (χ^2^_324_ = 427.614, *p* < 0.001; GFI = 0.89; NFI = 0.87; CFI = 0.97; RFI = 0.85; IFI = 0.97; TLI = 0.96; RMSEA = 0.031). The results of the multi-group analysis are presented in Table 5. The difference in the Chi-square of each path was calculated to test the moderating role of the facilitators’ service. Figure 2 illustrates which dimensions of hospitals’ service quality significantly affect consumers’ attitude and satisfaction toward cosmetic surgery tourism service along with the level of facilitators’ service quality.

In the case of the high facilitators’ service quality group, hospital tangibles and empathy significantly affected customers’ attitudes toward the service (β = 0.478; β = 0.367). On the contrary, for the low facilitators’ service quality group, only assurance significantly affected the attitude toward service (β = 0.572). As shown in the result of SEM, reliability, and responsiveness had no significant effects on customers’ attitudes toward service in both the groups. According to the Chi-square test, there were significant differences in the effect of tangibles (*p* = 0.003) and assurance (*p* = 0.035) on the attitudes toward the service between the two groups. Specifically, for the service quality group considering the high facilitators, the effect of hospitals’ tangibles on customers’ attitudes toward services is greater than that of the low facilitators. In contrast, when the facilitators’ service quality is low, hospitals’ assurance significantly affected their attitude toward tourism service. In this regard, H7 was partially supported. Even though there exists invariance at the model level, the effect of hospitals’ reliability, responsiveness, and empathy on the attitude toward medical tourism did not show significant differences along the level of service quality of the facilitating agents.

## 5. Discussion

As a core service provider for cosmetic surgery in the context of medical tourism, hospitals indicated positive effects on attitude and satisfaction via three dimensions of SERVQUAL in this study [23]. Specifically, the result of SEM indicates that tangibles, assurance, and empathy are essential components of cosmetic hospitals’ service quality, which significantly affect customers’ attitudes toward cosmetic surgery in medical tourism. SEM also showed that customers’ attitude toward cosmetic surgery significantly affects their satisfaction in the context of the service received.

Chinese cosmetic surgery medical tourists presented the five dimensionalities of SERVQUAL in the measurement model analyzing hospital service quality; however, reliability and responsiveness presented no significant effect on attitude. This is because these factors are considered to be relatively less important in influencing the performance of the medical industries, such as in the case of medical tourism for cosmetic surgery. Patients seeking cosmetic surgery require a particularly high technical quality compared to other surgical situations. Most importantly, the consumers of cosmetic surgery want the outcomes of their surgeries to be successful [73]. In this respect, the indicators that can predict the technical quality are considered as the first-order components that determine service quality. Therefore, assurance representing the trustworthiness of surgeons and staff, and tangibles enabling cutting-edge technical support to require primary focus. However, empathy is regarded as a relatively less important dimension of SERVQUAL, which when compared to reliability and responsiveness in general service industries is not related to medical service and therefore showed no significant effect. These results reflect on the industrial characteristics of medical tourism and are related to a high level of satisfactory experience that determines the expectation level of patients. According to the empirical studies conducted in the hospitals of the US and Korea, empathy affects service satisfaction and loyalty beyond the other dimensions of SERVQUAL [74,75]. Consistent with these findings, empathy shows a positive effect on attitude toward cosmetic surgery tourism service, whereas reliability and responsiveness do not.

The results of this study show how the service quality of the two entities interacts in the supply chain of medical tourism for cosmetic surgery, under the collaboration of hospitals and facilitators, affecting the attitude and satisfaction of consumers. Although consumers assess the facilitator’s service quality holistically without considering multiple dimensionalities, they indicate a nested service provision by moderating the influence of the service dimension of the core service provider. Further, this moderation effect influences hospitals’ tangibles and assurance on customers’ attitudes toward service. Consumers using cosmetic surgery medical tourism services determine their attitudes according to hospitals’ service quality with respect to tangibles and empathy dimensions when they perceive high service quality to facilitating agents, while they focus on hospital assurance when combined with low-quality facilitating agents. Throughout the analysis, we indicate that the criteria for evaluating hospitals, the main subjects of cosmetic surgery services, may vary depending on the role of facilitating agents in cosmetic surgery medical tourism.

## 6. Conclusions

### 6.1. Theoretical Contribution

The results indicating the positive effects of service quality on customers’ attitudes toward the incorporated service are consistent with previous studies [76]. Furthermore, the positive effect of attitudes on satisfaction has confirmed the findings of Boulding et al. in the context of cosmetic tourism [64]. Distinctively, the current study proves that there are three representative dimensions of hospitals’ service quality affecting customers’ attitudes toward cosmetic surgery medical tourism: tangibles, assurance, and empathy.

The primary contribution of this study is to contribute to the existing literature on medical tourism by developing a consumer evaluation model that reflects on the collaborative service structure between core and peripheral service providers. Previous studies on medical tourism have considered the influence of facilitators on consumers’ attitude and satisfaction toward incorporated service from the additive perspective of a core service provider. The quality of the interaction between the collaborative partners was considered by independently including variables such as cooperation in the research model [77]. This study initially proposes to suggest and test the possibility of the relative importance of the dimension of SERVQUAL in exhibiting a change. Similarly, the results of this study have a theoretical significance in that the existing service marketing literature on the service supply chain. This suggests that to understand the assessment of consumer’s incorporated service, a more rigorous consideration of the service attributes and quality is required in which the interactions between collaborative partners are examined.

### 6.2. Practical Contribution

Based on the results of this study, hospitals’ managers should focus more on the dimensions of tangibles, assurance, and empathy because these three factors positively enhance customers’ attitudes toward incorporated service, which in turn causes higher satisfaction. Simultaneously, managers need to recognize the role of facilitating agents. The service quality of facilitating agents lowers the impact of hospitals’ assurance while reinforcing the impact of hospitals’ tangibles. An inefficient alliance with the facilitating agents can cause the service quality of hospitals to affect the evaluation of the overall cosmetic surgery medical tourism service negatively. Therefore, managers should consider the effect of facilitators’ service quality when developing marketing strategies. In addition, hospitals should coordinate and keep in touch with facilitating agents to contribute to the success of international medical tourism.

Medical tourism for cosmetic surgery can be seen as the collaborative form of two separate services: cosmetic surgery and tourism. Although this type of tourism has a specific purpose, customers’ consumption behavior may diversify according to the role of the facilitating agent in addition to the purpose of their visit. To explain this service incorporation, identifying the service quality dimension of each entity according to the traditional SERVQUAL approach and investigating the effect of each dimension on attitude and satisfaction will serve as a guideline for future research related to incorporated services. Moreover, as the rate of spread of culture increases rapidly, consumers who follow popular beauty standards in other countries are likely to realize their needs for cosmetic surgery tourism services. In accordance with these environmental changes, defining and preparing service dimensions for foreign consumers to be strengthened by each entity imply that the competitiveness between countries and comparative advantage over competitors within the country can be obtained. Therefore, it is essential to realize and reflect on strengthening the service quality dimensions that may consequently change through the role of facilitating agents.

## 7. Limitations and Recommendations

The scope of this study is relatively limited because it only focuses on Korean hospitals and the cosmetic surgery sector of the medical industry. Therefore, it may not present a comprehensive picture of medical tourism. Future research could be conducted on a variety of sectors that involve medical tourism to compare the variation in the effects of the different sectors. Second, this study fully adopts the SERVQUAL method, which is simple and well established in the existing literature. With the development of technology and the internet, there are many extended service quality measurements, such as E-S-QUAL or M-S-QUAL [78,79]. Moreover, e-word-of-mouth (eWOM) has emerged as a new phenomenon that is similar to that of traditional word-of-mouth but provides more convenience, anonymity, and a variety of communication tools regardless of time and space. In addition, the rapid growth of online communication platforms and the diffusion of two-way interactions among customers with regard to products and services have transformed online notifications, reviews, opinions, and recommendations into sources of opportunities and challenges [80]. Therefore, eWOM can affect the impact of service quality on customers’ attitudes. Future studies could examine the moderating effect of eWOM on the effects of service quality on customers’ attitudes toward services provided. Third, this study has a limitation in that it measured the immediate satisfaction of patients for medical tourism. However, medical tourism combined with cosmetic surgery is more appropriate to measure satisfaction with long-term experience. To supplement this point, future research needs to identify factors that can affect the satisfaction with long-term experience of cosmetic surgery medical tourism by gathering longitudinal data. Moreover, long-term observation of the entire industry is required to understand the Chinese patients’ and behavioral patterns that decide on tourism to Korea for cosmetic surgery. Finally, this study put less consideration for the effects of various types of cosmetic surgery or the motivation for cosmetic surgery. Depending on the type of cosmetic surgery or individual motivation, the dimensions of service quality that patients evaluate importantly can be shifted, which can change the underlying mechanism for assessing satisfaction. Although this study relatively paid more attention to the interaction between the hospital and the facilitator, it is necessary to explore the moderating effect due to the patient’s individual variables in future research.

## Figures and Tables

**Table 1:** Participants’ socio-demographic characteristics.

Variable	Group	N (%)
Gender	Male	78 (23.4)
	Female	245 (73.4)
	No response	11 (3.3)
	Total	334 (100.0)
Age	17 years or less	4 (1.2)
	18–29 years	103 (30.8)
	30–39 years	119 (35.6)
	40–49 years	71 (21.3)
	50–59 years	27 (8.1)
	60–69 years	5 (1.5)
	70 years or more	0 (0.0)
	No response	5 (1.5)
	Total	334 (100.0)
Annual Income	Less than $25,000	43 (12.9)
	$25,000~$39,999	101 (30.2)
	$40,000~$54,999	97 (29.0)
	$55,000~$69,999	47 (14.1)
	$70,000~$84,999	22 (6.6)
	$85,000~$99,999	11 (3.3)
	$100,000 or more	5 (1.5)
	No response	8 (2.4)
	Total	334 (100.0)
Marital Status	Married	159 (47.6)
	Divorced	33 (9.9)
	Widowed	24 (7.2)
	Never married	113 (33.8)
	No response	5 (1.5)
	Total	334 (100.0)

**Table 2:** Result of Exploratory Factor Analysis for Hospital’s SERVQUAL.

Factors (% Variance Explained; Eigen Value)	Service Quality Attributes	Factor Loading	Cronbach’s Alpha
Tangibles (14.824%; 2.965)	The hospital has up-to-date equipment.	0.771	0.865
	The hospital’s physical facilities are visually appealing.	0.762	
	The hospital has clean and comfortable rooms.	0.660	
	Materials (including leaflets) associated with the hospital are easily understood.	0.576	
Reliability (13.900%; 2.780)	The hospital provides its services at the time it promises to do so.	0.545	0.860
	The hospital staff are dependable.	0.671	
	The hospital staff’s record-keeping is accurate.	0.697	
	The hospital is well organized.	0.645	
Responsiveness (10.875%; 2.175)	The hospital staff tell the patients exactly when the services will be performed.	0.733	0.824
	Patients receive prompt service from the hospital staff.	0.753	
Assurance (17.481%; 3.496)	Patients feel safe in their interactions with the hospital staff.	0.613	0.895
	The hospital staff are knowledgeable.	0.682	
	Staffs get adequate support from the hospital to perform their jobs well.	0.709	
	Patients trust the Dr. & the staff.	0.614	
	The hospital staff thoroughly explain a patient’s condition.	0.580	
	The behavior of the hospital staff instills confidence in customers.	0.610	
Empathy (14.199%; 2.840)	The hospital has the patients’ best interests at heart.	0.648	0.844
	The hospital has operating hours convenient to all patients.	0.707	
	Patients are waiting for a short time.	0.821	
	The hospital staff understand your specific needs.	0.568	

Note. KMO measure = 0.962; Bartlett’s test: χ^2^_190_ = 4412.821, *p* < 0.001.

**Table 3:** Result of CFA.

Variable	Standardized Loading	AVE	MSV	Composite Reliability
Hospital Tangibles	0.83 0.80 0.75 0.77	0.62	0.57	0.87
Hospital Reliability	0.73 0.81 0.82 0.77	0.61	0.58	0.86
Hospital Responsiveness	0.83 0.84	0.70	0.51	0.82
Hospital Assurance	0.77 0.73 0.73 0.78 0.76 0.78	0.58	0.58	0.89
Hospital Empathy	0.72 0.70 0.77 0.84	0.57	0.55	0.84
Attitude toward Service	0.86 0.87 0.79 0.69	0.64	0.50	0.88
Service Satisfaction	0.92 0.86	0.79	0.50	0.88

Goodness-of-fit: χ^2^_277_ = 400.853, *p* < 0.001; GFI = 0.92; NFI = 0.94; CFI = 0.98; RFI = 0.92; IFI = 0.98; TLI = 0.98; RMSEA = 0.037.

**Table 4:** Result of SEM.

Structural Path	β	*t*-Value
Hospital Tangibles → Attitudes toward Service	0.292	2.480 *
Hospital Reliability → Attitudes toward Service	−0.130	−0.837
Hospital Responsiveness → Attitudes toward Service	−0.042	−0.384
Hospital Assurance → Attitudes toward Service	0.415	2.253 *
Hospital Empathy → Attitudes toward Service	0.275	2.148 *
Attitudes toward Service → Satisfaction on Service	0.808	15.501 ***

Note: *p* * < 0.05, *p* ** < 0.01, *p* *** < 0.001. Goodness-of-fit: χ^2^_282_ = 429.774, *p* < 0.001; GFI = 0.91; NFI = 0.93; CFI = 0.98; RFI = 0.92; IFI = 0.98; TLI = 0.97; RMSEA = 0.040.

**Table 5:** Result of multi-group analysis.

KERRYPNX	High Facilitator’s Service Quality (*n* = 162)		Low Facilitator’s Service Quality (*n* = 172)			
Structural Path	β	*t*-Value	β	*t*-Value	Δχ^2^	*p*-Value
HT → ATT	0.478	3.427 ***	0.018	0.117	8.758	0.003 **
HRL → ATT	−0.189	−1.054	0.174	0.795	1.759	0.185
HRS → ATT	−0.061	−0.396	−0.056	−0.377	0.014	0.906
HA → ATT	0.087	0.420	0.572	2.375 *	4.454	0.035 *
HE → ATT	0.367	2.683 **	0.065	0.288	1.072	0.300
ATT → SS	0.680	7.084 ***	0.747	9.564 ***	0.256	0.613

Note: *p* * < 0.05, *p* ** < 0.01, *p* *** < 0.001. Goodness-of-fit: χ^2^_324_ = 427.614, *p* < 0.001; GFI = 0.89; NFI = 0.87; CFI = 0.97; RFI = 0.85; IFI = 0.97; TLI = 0.96; RMSEA = 0.031. HT: Hospital Tangibles; HA: Hospital Assurance; HE: Hospital Empathy; HRL: Hospital Reliability; HRS: Hospital Responsiveness; ATT: Attitude toward Service; SS: Satisfaction on Service.

## Data Availability

The data presented in this study are available on request from the corresponding author.

## Abbreviations

EFA	Exploratory Factor Analysis
CFA	Confirmatory Factor Analysis
AVE	Average Variance Extracted
RMSEA	Root Mean Square Error of Approximation
MSV	Maximum Shared squared variance
CR	Composite Reliability
GFI	Goodness-of-Fit Index
NFI	Normed Fit Index
CFI	Comparative Fit Index
RFI	Relative Fit Index
IFI	Incremental Fit Index
TLI	Tacker–Lewis fit Index
KMO index	Kaiser–Meyer–Olkin index

## Appendix A. Measurement Items

**Table A1:** Measurement Items.

**Construct**	**Measurement Items**	**Source**
Hospital Tangibles	HT 1. The hospital has up-to-date equipment. HT 2. The hospital’s physical facilities are visually appealing. HT 3. The hospital has clean and comfortable rooms. HT 4. Materials (including leaflets) associated with the hospital are easily understood.	Chakravarty (2011); Guiry and Vequist (2011); Guiry et al. (2013); Parasuraman et al. (1988)
Hospital Reliability	HRL 1. The hospital provides its services at the time it promises to do so. HRL 2. The hospital staff are dependable. HRL 3. The hospital staff keep accurate records. HRL 4. The hospital is well organized.	
Hospital Responsiveness	HRS 1. The hospital staff tell the patients exactly when services will be performed. HRS 2. Patients receive prompt service from the hospital’s staff.	
Hospital Assurance	HA 1. Patients feel safe in their interactions with the hospital’s staff. HA 2. The hospital staff are knowledgeable. HA 3. Staff gets adequate support from the hospital to perform their jobs well. HA 4. Patients trust the doctors and staff. HA 5. The hospital staff thoroughly explain a patient’s condition. HA 6. The behavior of the hospital staff instills confidence in customers.	
Hospital Empathy	HE 1. The hospital has the patients’ best interests at heart. HE 2. The hospital has operating hours convenient for all patients. HE 3. Patients have to wait for a short time. HE 4. The hospital staff understand your specific needs.	
Facilitator Tangibles	FT 1. The facilitator has up-to-date equipment and technology. FT 2. Staff of the facilitator are well dressed and of neat appearance. FT 3. The physical facilities at the facilitator are visually appealing. FT 4. Materials associated with the facilitator’s service (such as pamphlets or quotations) are visually appealing in the travel agency.	
Facilitator Reliability	FRL 1. When the facilitator promises to do something by a certain time, it will do so. FRL 2. The facilitator insists on error-free records. FRL 3. The facilitator provides a convenient location and contact with the tour, hotel, and hospital escorts. FRL 4. The facilitator keeps to all patient schedules.	
Facilitator Responsiveness	FRS 1. Staff of the facilitator tell patients exactly when services will be performed. FRS 2. Staff of the facilitator are always willing to help patients.	
Facilitator Assurance	FA 1. The behavior of the staff of the facilitator instills confidence in patients. FA 2. Patients of the facilitator feel safe in their transactions. FA 3. Staff of the facilitator are consistently courteous with patients. FA 4. Staff of the facilitator have the required knowledge to answer patient questions.	
Facilitator Empathy	FE 1. The facilitator gives patients individual attention. FE 2. The facilitator has operating hours convenient for all its patients. FE 3. The facilitator has the patients’ best interests at heart. FE 4. The staff of the facilitator understand the specific needs of their patients.	
Attitude toward medical tourism service	ATT 1. My attitude toward medical travel services is positive/negative. ATT 2. My attitude toward medical travel services is good/bad. ATT 3. My attitude toward medical travel services is beneficial/harmful. ATT 4. My attitude toward medical travel services is wise/foolish.	Ellen et al. (2000)
Satisfaction on medical tourism service	SAS 1. I was satisfied with the medical travel services provided. SAS 2. I was delighted with the medical travel services provided.	Voss et al. (1998)
